# Assessing patient preferences for medical decision making - a comparison of different methods

**DOI:** 10.3389/fdgth.2025.1641765

**Published:** 2025-11-13

**Authors:** Jakub Fusiak, Andreas Wolkenstein, Verena S. Hoffmann

**Affiliations:** 1 Institute for Medical Information Processing, Biometry, and Epidemiology, Ludwig-Maximilians-Universität München, Munich, Germany; 2 Institute of Ethics, History and Theory of Medicine, Ludwig-Maximilians-Universität München, Munich, Germany

**Keywords:** patient preference, shared decision making, medical decision aids, potentially all pairwise rankings of all possible alternatives (PAPRIKA) method, questionnaire method

## Abstract

**Background:**

Patient preferences are a critical component of shared decision-making (SDM), particularly when choosing between treatment options with differing risks and outcomes. Many methods exist to elicit these preferences, but their complexity, usability, and acceptance vary.

**Objective:**

We aim to gain insight into the acceptance, effort and preferences of participants regarding five different methods of preference assessment. Additionally, we investigate the influence of health status, experiences within the health system and of demographic factors on the results.

**Methods:**

We conducted a cross-sectional online survey including five preference elicitation Methods: best-worst scaling, direct weighting, PAPRIKA (Potentially All Pairwise Rankings of all Possible Alternatives), time trade-off, and standard gamble. The questionnaire was distributed via academic and patient advocacy mailing lists, reaching both healthy individuals and those with acute or chronic illnesses. Participants rated each method using six standardized statements on a 5-point Likert scale. Additional items assessed general acceptance of algorithm-assisted preference assessments and the clarity of the questionnaire.

**Results:**

Of 258 initiated questionnaires, 123 (48%) were completed and included in the analysis. Participants were diverse in age, gender, and health status, but predominantly highly educated and digitally literate. Across all measures, the PAPRIKA method received the highest ratings for clarity, usability, and perceived ability to express preferences. Simpler methods (best-worst scaling, direct weighting) were rated as less useful for capturing nuanced preferences, while abstract utility-based methods (standard gamble, time trade-off) were seen as cognitively demanding. Subgroup analyses showed minimal variation across demographic groups. Most participants (82%) could imagine using at least one of the presented methods in real clinical settings, but also emphasized the importance of physician involvement in interpreting results.

**Conclusion:**

The interactive PAPRIKA method best balanced cognitive demand and expressiveness and was preferred by most participants. Structured methods for preference elicitation may enhance SDM when integrated into clinical workflows and supported by healthcare professionals. Further research is needed to evaluate their use in real-world decisions and among more diverse patient populations.

## Introduction

The number of prognostic and predictive models aiming to inform on treatment decisions has been steeply rising within the last two decades (1). Many of them successfully use traditional and advanced statistical methods, which also include machine learning and artificial intelligence, to allow health care professionals (HCP) and patients to better estimate the patient's prognosis and potential outcome of different therapies (2).

While on the one hand the use of algorithms could lead to a mechanization within the relationship between HPC and the individual patient, on the other hand these algorithms could also be an opportunity to strengthen shared decision-making (SDM) (3, 4). A plethora of studies have shown that SDM is a key component of high-quality healthcare (5–9).

SDM is considered the final step completing the implementation of evidence-based medicine (10). It relies on understanding patient values and preferences and including them into the decision process (11). Patient values and preferences are the unique understandings, preferences, concerns, expectations and life circumstances of each patient (12). *Values* are defined as a patient's attitudes and perceptions about certain healthcare options, and *preferences* are their preferred choices after accounting for their values (13).

These preferences have to be assessed. While there is research on what patients value in healthcare (14) and qualitative evidence on how patient preferences are integrated into healthcare (11) questions to elicit patient preferences can also be included either directly into algorithms that inform treatment decisions or within the process of applying the algorithm within the clinical workup. This way these preferences receive the same attention as other clinical factors and it would become part of the routine to assess them in a structured manner.

To achieve this benefit, patient preferences need to be assessed in a valid and approachable way to truly reflect a patient's values. Many methods for the assessment of preferences are available from decision theory, but they need to be adapted to the medical context.

After finalizing a scoping review on how patient preferences have been incorporated into medical decision algorithms and models (15, 16) we chose five assessment methods for patient preferences to further investigate: Best-worst scaling (17), direct weighting (18), Potentially All Pairwise Rankings of all possible Alternatives (PAPRIKA) (19), time trade-off (20) and standard gamble (21). The selection of the methods reflects the diversity of approaches and cover a wide spectrum of complexity and cognitive demand.

Direct Weighting (DW) and Best-Worst Scaling (BWS) are methods for attribute-weighting the importance of treatment attributes. In DW patients directly attribute weights to given criteria, e.g., by distributing points adding up to 100. DW is an intuitive approach in clinical research and quality-of-life studies (18). It has also been used for prostate cancer screening decision analysis tool (22). BWS was developed in mathematical psychology (17) and has widespread in marketing (23), social care (24), health preferences research (25) and more. In BWS criteria are split into small sets. Patients then chose the most and least important options from these sets. This generates relative importance scores to rank items and estimate preference weights. BWS can, for example, be included in decision aid for psychiatry treatment choices (26).

Standard Gamble (SG) and Time Trade-off (TTO) qualify preferences by using the assessment of individual health utility. SG measures preferences under uncertainty. Patients are asked to choose between living in a certain health state for sure, or taking a risky treatment with a probability *p* of full health and (1-p) immediate death. By varying *p* until the patient is indifferent between the certain health state and the probability of immediate death (1-p) the utility value of the health state is derived on a scale from 0 (equivalent to death) and 1 (equivalent to perfect health) (21). This method was for example used in a decision aid for prostate cancer treatment (27). The TTO method measures how much lifetime in full health is considered equivalent to a longer time in a less desirable state by the patient (20). Simes et al. applied TTO to statistical decision theory with utility elicitation (28). Both methods originated in health economics (29), and are widely used for quality-of-life estimation (30) and health technology assessment (31). TTO is frequently used for value set construction (32, 33).

PAPRIKA (Potentially All Pairwise RanKings of all possible Alternatives), which infers criterion weights from a sequence of pairwise comparisons where scenarios differ on exactly two attributes (19). PAPRIKA is a relatively new method, developed in 2008 and patented in 2010 by the company 1,000 minds and has gained popularity in many domains, such as clinical (34) and guideline development (35) contexts because it aligns with multi-attribute treatment decisions and can provide transparent weighting structure.

Each method differs in cognitive burden, output (utilities vs. weights/rankings), and fit with clinical workflows (33, 36–38). While each of these methods can be used to assess patient preferences on an individual level, prior work suggests method acceptability can vary by patient population and context (38).

Within the EPAMeD project (Ethics and practice of algorithm-supported decision making in medicine) we are aiming to develop an algorithmic tool for decision support for patients with multiple sclerosis. Here, we describe the adaption of several methods for preference assessment to a hypothetical treatment decision scenario and the evaluation and acceptance of these methods within a group of patients and healthy persons.

## Aims and objectives

In this study, we compare a wide range of methods that vary in cognitive complexity and level of detail for eliciting preferences. These methods are presented to patients with different chronic or acute health conditions, as well as to healthy individuals.

We aim to gain insight into the acceptance, effort and preferences of participants regarding the different methods. Additionally, we investigate the influence of health status, experiences within the health system and of demographic factors on the results.

## Materials and methods

### Study design

This is a cross-sectional questionnaire study in volunteers conducted via an online survey platform over a one-month period.

### Questionnaire

The online questionnaire was in German language and consisted of 18 pages (Supplementary File S1). The phrasing reported here is an English translation. The first page described the topic and project. The second page assessed demographic data of the participant.

The third page introduced the hypothetical scenario. It asked participants to imagine they had a severe progressing disease which leads to severe limitations in mobility, need for care and early death when untreated. The time to progression however is very individual. There are three therapeutic options for the disease with different effectiveness, application routes and side effects. Participants are then asked to imagine they have to choose one treatment together with their treating doctor, who is using structured methods to support the decision.

The following pages presented the instances of the five investigated methods adapted to the hypothetical scenario and three treatment options with varying levels of the attributes: Effectiveness, side effects and application route.

In DW, participants indicated relative importance by allocating a fixed budget of points across the three attributes. Operationally, they were asked to distribute 100 points across the attributes (effectiveness, side effects and application route) so that the total summed to 100, placing more points on attributes that mattered more to them.

In BWS, respondents evaluated small sets of attributes and identified, within each set, the most important and the least important item. For example, given a set containing reduction of symptoms, avoiding side effects, and mode of administration, they selected one as most important, one as least important, and one as nether important nor not important.

In PAPRIKA, we presented pairwise comparisons between hypothetical treatments that differed on two attributes. For instance, contrasting an option with a higher effectiveness requiring monthly infusion against an option with a moderate effectiveness with daily oral pills. Participants could then indicate which option they preferred, or whether the methods were equally desirable.

For the utility-based methods, TTO elicited the point of indifference between living a longer in a less desirable health state and a shorter time in full health. Participants were asked, if they would rather live 10 years with a given combination of attributes representing a specific treatment regimen vs. 10-X years in full health. X was decreased by one year per iteration until indifference. This was performed for all three hypothetical treatment options.

SG elicited utilities by offering a choice between a certain health state and a risky treatment resulting in full health with probability *p* and immediate death with probability (1−*p*). Patients were asked if they want to provide a risk *p*. If the answer was yes, the participants were able to adjust the point of indifference.

By including methods that differ in their cognitive demands and ease of use, our study aims to identify approaches that balance methodological rigor with acceptability in different patient groups. Furthermore, the study predicts the practicality of these methods in real-world SDM contexts, including their ability to help patients to weigh treatment options and effectively express their preferences. After the presentation of each method, where participants could enter their choices, they were shown a page with six statements to rate the method on a 5-point Likert scale (Agree not at all - Fully agree). The statements regarding each method respectively were:

1. I was able to express which type of treatment I prefer.
2. I have thoroughly weighed the different aspects of a possible treatment using this method.
3. I find this method too complex.
4. Overall, I am satisfied with this method.
5. If my doctor uses this method to find a suitable treatment, I have the opportunity to actively contribute my preferences.
6. I hope this method will be used during consultations with my treating doctor.

Additionally, participants were asked to rate three statements regarding the acceptance and satisfaction with shared decision-making and the overall concept of algorithm-assisted assessment of patient preferences, as well as three statements regarding the clarity of the questionnaire. We again used a 5-point Likert scale (Agree not at all - Fully agree). The statements were:

1. It is important to me to be able to express my preferences in detail when choosing a therapy
2. The methods presented helped me become aware of my preferences
3. I would prefer to complete the preference assessment calmly at home rather than in a 15-minute conversation with my doctor
4. The questions in the survey were clear to me
5. There were words or expressions that I did not understand
6. There was enough context to be able to answer the questions

One more question was asked with yes and no as possible answers:

1. Can you imagine that your preferences for selecting a therapy could be assessed using one of the methods shown earlier?

Finally, there was an option to leave comments in free text. The full questionnaire (original version and English translation) is provided in Supplementary File S1.

To ensure feasibility and consistency we performed a pre-test of the questionnaire with ten participants. The pretest resulted into modifications mainly of wording and presentation of scales. Results of the pretest are not included in the data reported in this manuscript.

### Participants

To include a broad range of individuals—both healthy and those with chronic or acute conditions—we distributed our questionnaire via multiple channels: the informational newsletter of Ludwig Maximilian University (Munich, Germany), the mailing list of our Ph.D. Program in Epidemiology & Public Health, and the mailing list of *Selbsthilfezentrum München*, a patient advocacy organization. All recipients were motivated to further share the link in order to reach more and more diverse participants. While participants could choose not to answer single questions their responses were not included into the analysis if they did not follow through the questionnaire to the end.

The sample size calculation was based on the Friedman test. With a sample size of 100 participants, the estimated power to detect a difference of at least 0.5 points on the Likert scale between methods is approximately 93%.

### Analysis

Data collected on nominal or ordinal scale is reported using percentages, responses collected on the Likert scale are reported by calculating the means and standard deviations (sd). Depending on group sizes, results are also reported in subgroups according to demographic data. To identify relevant differences the Friedman tests and Wilcoxon tests were used. However, due to the explorative nature of this study we are not formally setting a level of significance. Spearman correlation coefficients were calculated to identify correlations between Likert scales and demographic data.

Comments were analyzed using thematic analysis following the approach described by Braun and Clarke (39). Two researchers (JF and VSH) independently reviewed all comments, conducted open coding, and iteratively developed a set of themes that captured recurring patterns in the data. Discrepancies were discussed until consensus was reached.

### Software

The questionnaire was developed using REDCap Version 14.6.7 and provided on the servers of the Institute for Medical Information Processing, Biometry and Epidemiology, LMU, Munich.

All statistical analyses were performed using R version 4.3.1.

## Results

The questionnaire was online for one month between February 5th, 2025 and March 4th, 2025. The questionnaire was started 258 times, 123 participants completed the questionnaire (48%).

### Demographics and personal information

The majority of participants was female (65%), regarding age the participants were distributed almost evenly between four age groups between 18 and 75 years, only two participants were older than 75 years. The majority of participants was fluent in German (98%) and held a high school (29%) or university diploma (60%). Almost all participants except for one stated to be using a computer or smartphone daily. 80% of participants had consulted a doctor within the last three months, 55% reported to be chronically ill, with 21% (*n* = 26) owning a disability certificate (German “Schwerbehindertenausweis”) with a median grade of disability of 50% (range 40%–100%). Detailed results are reported in Table 1.

**Table 1:** Demographic data and personal information.

Characteristic	*n*	%
Gender		
Female	80	65
Male	42	34
Diverse	1	1
Age		
18–30 years	31	25
31–45 years	30	24
46–60 years	33	27
61–75 years	27	22
>75 years	2	2
Fluent in German (yes)	121	98
Highest education		
None	0	0
Elementary School	0	0
“Hauptschule”	4	3
“Mittlere Reife”	11	9
“Hochschulreife”	35	29
University degree	73	59
Computer/Smartphone use		
Daily	122	99
At least once per week	1	1
At least once per month	0	0
Less than once per months	0	0
Time since last doctor's visit		
Less than 3 months ago	98	80
3–6 months ago	17	14
6–12 months ago	3	2
More than 12 months ago	5	4
Self-description of health status		
Very good	15	12
Good	59	48
Medium	37	30
Poor	9	7
Very poor	3	2
Current diseases		
Yes, acute	7	6
Yes, chronic	68	55
No	48	39
Owning a disability certificate (yes)	26	21
Degree of disability		
≤50%	13	50
>50%	11	42
Information not provided	2	8

### Evaluation of the five presented methods

The participants were shown five methods which were adapted to the hypothetical scenario. After the presentation of each method they were asked to evaluate the method by agreeing or disagreeing with six statements on a 5-point Likert scale.

The results show a clear preference of the participants for the PAPRIKA method. PAPRIKA was rated better regarding five statements with similar or smaller standard deviation than the other methods.

The standard gamble and time trade-off methods received very similar ratings regarding all statements, which were worse than the PAPRIKA ratings but better than the ratings for direct weighting and best-worst scaling. Ratings for these very simple methods were worse for all statements except for the statement regarding complexity. Table 2 shows the mean Likert scores for each statement by method.

**Table 2:** Comparison of results between the five methods.

Question	PAPRIKA	Direct weighting	Best-worst scaling	Standard gamble	Time trade-off
I was able to express which type of treatment I prefer.	4.0 ± 0.9	3.3 ± 1.3	3.1 ± 1.2	3.6 ± 1.2	3.5 ± 1.3
The methods presented helped me become aware of my preferences.	3.8 ± 1.1	3.0 ± 1.3	2.8 ± 1.2	3.4 ± 1.2	3.3 ± 1.3
I find this method too complex.	2.1 ± 1.2	1.7 ± 1.1	1.6 ± 0.9	2.1 ± 1.1	2.1 ± 1.1
Overall, I am satisfied with this method.	3.5 ± 1.1	2.9 ± 1.2	2.7 ± 1.3	3.1 ± 1.3	3.0 ± 1.2
If my doctor uses this method to find a suitable treatment, I have the opportunity to actively contribute my preferences.	3.9 ± 1.0	3.2 ± 1.2	2.9 ± 1.2	3.4 ± 1.2	3.3 ± 1.2
I hope this method will be used during consultations with my treating doctor.	3.8 ± 1.1	3.0 ± 1.4	2.8 ± 1.3	3.2 ± 1.3	3.1 ± 1.1

Reported are means and standard deviations from the Likert scale (range 1–5/Agree not at all - Fully agree).

The Friedman test showed that the differences between methods were very large (*p* < 0.0001 for each statement). *Post-hoc* comparisons indicated that PAPRIKA differed substantially from all other methods for almost all statements.

### Subgroup analyses

There were no relevant differences between genders regarding the evaluation of methods. Only male and female participants were compared due to the small number of diverse participants (*n* = 1).

In general, the age group 46–60 years evaluated the methods more critical than the other three age groups. For PAPRIKA differences between the overall mean and the age group means were rarely larger than ±0.5 points on the Likert scale. Older participants rated the more parsimonious methods (Direct Weighting and Best-worst Scaling) generally better than younger participants. The Spearman correlation coefficient between age groups and Likert scale results of the single statements showed low to moderate correlations between −0.4 and +0.4. Only the correlation between age and the wish to use Direct Weighting for future treatment decisions was higher (0.44).

Overall, the mean ratings on the Likert scale for the different methods did not vary substantially across the self-reported health status. Spearman correlations did not exceed ± 0.2. The only exception was the question of whether participants found the method too complex, which was generally rated higher by those who reported poor or very poor health.

Only very small differences could be detected between the mean Likert scale ratings of participants with or without chronic diseases. Participants who reported to own a disability certificate on the other hand rated all suggested methods better than participants without a disability certificate.

Very few participants reported having visited a doctor more than six months ago (*n* = 8). No relevant differences in the mean rating of methods were observed between participants who visited a doctor up to three months or three to six months ago.

Only 15 participants did not receive higher education. If a participant had a university degree or not was strongly correlated to the age group, indicating that many students took part in the study. Overall, this made a subgroup analysis of the level of education uninformative.

Due to the small numbers of participants who did not speak German fluently (*n* = 2) and who did not use a smartphone or computer daily (*n* = 1) we did not perform subgroup analyses on those.

### Overall evaluation of algorithm-assisted assessment of patient preferences

Following the presentation and rating of the five methods we asked the participants some general questions regarding their acceptance and satisfaction with the overall concept of algorithm-assisted assessment of patient preferences. Full results are shown in Table 3.

**Table 3:** Results of the overall evaluation of algorithm-assisted assessment of patient preferences and of the questions regarding the clarity of the questionnaire.

Statements	Points on the Likert scale					Mean (sd)
	1	2	3	4	5	
It is important to me to be able to express my preferences in detail when choosing a therapy.	0	0	2	30	91	4.7 (0.5)
The methods presented helped me become aware of my preferences.	1	12	21	52	33	3.8 (1.1)
I would prefer to complete the preference assessment calmly at home rather than in a 15-minute conversation with my doctor.	8	14	38	36	27	3.5 (1.1)
The questions in the survey were clear to me.	9	7	13	35	59	4.0 (1.2)
There were words or expressions that I did not understand.	105	10	2	4	2	1.3 (0.8)
There was enough context to be able to answer the questions.	5	10	23	44	41	3.9 (1.1)

Most participants agreed strongly with the statement “It is important to me to be able to express my preferences in detail when choosing a therapy.” (mean 4.7, sd 0.5).

Most participants agreed with the statement “The methods presented helped me become aware of my preferences” (mean 3.8, sd 1.1).

Also, the statement “I would prefer to complete the preference assessment calmly at home rather than in a 15-minute conversation with my doctor.” was rather agreed to by the majority of patients (mean 3.5, sd 1.1).

The question “Can you imagine that your preferences for selecting a therapy could be assessed using one of the methods shown earlier?” was answered with “yes” by 82% (*n* = 101) of the participants.

### Clarity of the questionnaire

As one way of validation we asked three questions to confirm the questionnaire was understandable to the participants. Most participants agreed to the statements about clarity of the questions (mean 4.0, sd 1.2) and sufficiency of context (mean 3.9, sd 1.1). Only few participants reported not to have understood words or expressions in the questionnaire (mean 1.3, sd 0.8). Full results are reported in Table 3.

### Comments

In the last section of the questionnaire participants had the opportunity to leave a free text comment. Many of them were insightful regarding either the participants experience with the questionnaire or attitudes toward the methods. Overall, 28 comments were made. Thematic analysis identified four overarching themes:

1. Involvement of a doctor: Seven participants found the methods presented helpful to support decision making but pointed out that for discussion and decision-making a doctor needs to be involved.
2. Struggling with abstraction in the scenario: Seven participants described that they struggled with the hypothetical scenario.
3. Perceived burden: Three participants stated that they found the time trade-off and standard gamble methods too theoretical or psychologically burdensome.
4. Balancing usefulness and complexity: Two participants described PAPRIKA as useful but challenging as comparisons have to be read closely to identify the respective differences.

Nine participants left other comments containing singular topics like own experiences, confusion about specific methods (e.g., TTO), the level of detail in the information provided, or the wish for prevention instead of treatment.

## Discussion

Our study aimed to compare a variety of methods to assess patient preferences for therapy selection. The methods were presented to patients with different chronic or acute health issues as well as healthy individuals. With our study setup we reached healthy as well as chronically ill individuals and also a small number of participants with an acute disease. The participants were diverse regarding age, gender and health status but rather homogeneous regarding education and digital literacy.

Across all aspects we assessed, the participants showed a strong preference for the PAPRIKA method. The simpler methods (direct weighting and best-worst scaling) were perceived as too restrictive regarding the expression of individual preferences and balancing different aspects of the treatment options.

The more abstract methods (standard gamble and time trade-off) were rated better than the simple methods but worse than PAPRIKA. The comments hint that the abstract methods were perceived as too theoretical and too mentally burdensome as these methods focus on a trade-off in life years.

Obviously, these kinds of tools to aid shared decision making are not made for all patients and all healthcare situations. From a clinical perspective there needs to be sufficient time to make a shared decision. The presented methods are not meant to be applied in emergency situations. Also, the decision needs to be preference-sensitive, meaning there need to be two or more treatment options that are studied well enough to provide adequate information on likely treatment outcome and adverse event rates. This is often the case in chronic diseases with variable prognoses as multiple sclerosis and Parkinson's disease (40).

For patients who are comfortable using technology and prefer a more structured kind of decision making our study shows that a tool like PAPRIKA is the most agreed to. It needs to be determined if structured methods to assess patient preferences are also accepted by patients with a lower level of formal education who are as well proficient using digital tools. This group was underrepresented in our sample likely due to our ways of distributing our questionnaire.

Up until now in the medical field the PAPRIKA method has been mainly used for patient prioritization (34, 41, 42), medical research (43–45), health technology prioritization (46–48) and to inform public health decisions (49, 50). Applications directly including patients into their therapy decision do currently not exist on an individual level or stay theoretical (51).

Developing a patient preference assessment tool for a specific treatment decision according to our results must consider two major challenges:

1. It must enhance SDM. This could be validated using the SDM-Q-9 questionnaire. This questionnaire is made to measure level of SDM perceived by patients (52). It is regularly used (53) and translated into many languages (54–56). Future studies should assess whether using a method like PAPRIKA leads to higher SDM-Q-9 scores, indicating an improved shared decision process.
2. If therapeutic characteristics are presented, they must be based on the highest quality of evidence available. A tool like PAPRIKA which is modeled to assess patient preferences for a specific health care situation, needs to include high quality information e.g., on relapse rates or probability of adverse events to be helpful.

Both aspects make it inevitable to closely tie tools for assessing patient preferences into the clinical workflow. They cannot be stand-alone. They need to be introduced to the patients by healthcare professionals and the results need to be discussed with healthcare professionals. This is also a result of our study: While a majority of participants would agree to use structured methods for the assessment of their preferences and found them helpful to become aware of their own preferences there was a strong urge to discuss the results with the treating doctor.

As an additional tool for assessing preferences especially for informing changes of therapeutic measures the integration of wearable biomonitors may enhance the quality of therapy-related decision-making. Continuous, objective data on parameters such as activity, sleep, or physiological stress can provide patients with a more concrete understanding of how their condition and potential treatments affect daily life.

The presented methods can also be included into existing prognostic and predictive models ensuring that recommended treatments and treatment outcomes align with the patient's preferences, providing increased patient satisfaction, treatment adherence, and better health outcomes.

## Limitations

Our study has two main limitations: The sample composition and the hypothetical nature of the scenario.

While our sample of participants was diverse in terms of sex, age, and health status, the majority was highly educated with strong digital literacy. Thus, we were not able to capture the opinions of less educated persons and those who might be less comfortable using digital tools and devices. This bias could mean that the methods, especially the more complex ones, may appear more acceptable in our sample than they would in a less educated population. Therefore, our findings might represent a best-case scenario of understanding and engaging with these tools.

A participant in our study is in many ways different from a specific patient who needs to weigh treatment options for a specific disease. Patients experience the symptoms and know about their severity and perseverance. They know to what extend the disease influences their day to day life and are thus better able to trade-off between symptoms and potential adverse events of a therapy. These layers of knowledge had to be substituted for the participants or imagined by the participants making the setup more complex and – although inevitable for the studied subject – limit generalizability.

Additionally, we did not attempt to determine which method produces the most accurate preferences or whether all methods yield similar preference rankings for an individual. Our focus was on user experience and acceptance.

## Conclusion

In conclusion, our study suggests that an interactive trade-off method (e.g., PAPRIKA) best achieved the balance of detail and usability needed for capturing patient preferences in medical decisions. Participants were willing and able to engage with such a tool, especially when they could do so on their own time, but still value physician guidance in interpreting the results. Future work should focus on integrating this approach into clinical workflows and testing it in more diverse patient populations and real decision-making scenarios.

## Data Availability

The raw data supporting the conclusions of this article will be made available by the authors, without undue reservation.
