# Supplementary material for: Assessing patient preferences for medical decision making - a comparison of different methods
Source: Front Digit Health. 2025 Nov 13;7:1641765. doi: 10.3389/fdgth.2025.1641765 (PMC12659191; doi:10.3389/fdgth.2025.1641765)
Supplement: Supplementary file 1 [file Datasheet1.pdf]

Bundesministerium für Bildung und Forschung

AAA

Liebe Teilnehmerinnen und Teilnehmer,

in unserem Forschungsprojekt wollen wir herausfinden, wie Patient/innen und Ärzt/innen am besten dabei unterstützt werden können, gemeinsam schwierige medizinische Entscheidungen zu treffen.

Auf den folgenden Seiten stellen wir Ihnen fünf Methoden vor und bitten Sie, diese Methoden anzuwenden. Nach jeder Methode haben Sie die Möglichkeit, diese anhand einiger Fragen zu bewerten.

Zusätzlich bitten wir Sie zu Beginn um einige Angaben zu Ihrer Person und am Ende um eine Gesamteinschätzung der Nützlichkeit dieser Methoden.

Die Umfrage ist anonym, Rückschlüsse auf Ihre Person können nicht gezogen werden.

Herzlichen Dank für Ihre Unterstützung!

Die Umfrage ist Teil des Projektes "EPAMeD - Ethik und Praxis der Algorithmus-unterstützten Entscheidungsfindung in der Medizin", über das Sie hier mehr erfahren können.
EPAMeD wird vom Bundesministerium für Bildung und Forschung (BMBF) gefördert (FKZ: 01GP2207).
Ansprechpartner für diese Umfrage ist Jakub Fusiak (jakub.fusiak@ibe.med.uni-muenchen.de).
Für datenschutzrechtliche Anliegen ist der Datenschutzbeauftragte der LMU verantwortlich (www.lmu.de/datenschutz).

Weiter

Save & Return Later

Bundesministerium
für Bildung
und Forschung

A A A

Bitte beantworten Sie uns zuerst folgende Fragen über Sie persönlich:

Alter:

* Pflichtfeld

- 18-30
- 31-45
- 46-60
- 61-75
- 76+

Geschlecht:

* Pflichtfeld

- Weiblich
- Männlich
- Divers

Sprechen Sie fließend deutsch?

* Pflichtfeld

- Ja
- Nein

Welcher ist der höchste Schulabschluss, den Sie erreicht haben?

* Pflichtfeld

- Ich habe keinen Schulabschluss
- Grundschulabschluss
- Hauptschulabschluss
- Mittlere Reife
- Allgemeine oder fachgebundene Hochschulreife
- Hochschulabschluss

Wie häufig nutzen Sie einen Computer oder ein Smartphone?

* Pflichtfeld

- Seltener als ein Mal pro Monat
- Mindestens ein Mal pro Monat
- Mindestens ein Mal pro Woche
- Täglich

Wann war Ihr letzter Arztbesuch?

* Pflichtfeld

- Vor weniger als 3 Monaten
- Vor 3 bis 6 Monaten
- Vor 6 Monaten bis 1 Jahr
- Vor mehr als 1 Jahr

Wie schätzen Sie selbst Ihren aktuellen Gesundheitszustand ein?

* Pflichtfeld

- Sehr gut
- Gut
- Mittel
- Schlecht
- Sehr schlecht

Haben Sie chronische oder akute Erkrankungen?

* Pflichtfeld

- Nein, keine
- Ja, akut
- Ja, chronisch

Haben Sie einen Schwerbehindertenausweis?

* Pflichtfeld

- Nein
- Ja

Überhaupt nicht Sehr viel

Haben Sie einen Schwerbehindertenausweis?

* Pflichtfeld

Nein

Ja

Falls Sie einen Behindertenausweis haben, wie hoch ist Ihr Grad der Behinderung?

	Überhaupt nicht				Sehr viel
**Erinnern Sie sich bitte an Ihre vergangenen Arztbesuche: In welchem Ausmaß konnten Sie dabei Ihre Präferenzen einbringen, als es um die medizinische Entscheidung ging?** * Pflichtfeld	○	○	◉	○	○

Zurück

Weiter

Save & Return Later

Bundesministerium
für Bildung
und Forschung

Szenario "Medizinische Behandlungsentscheidung"

Bitte stellen Sie sich vor, dass Sie eine schwere Krankheit haben, die sich mit der Zeit verschlimmert. Ohne Behandlung wird diese Krankheit dazu führen, dass Sie sich kaum noch bewegen können, auf Pflege angewiesen sind und früher sterben. Wie lange es dauert, bis sich die Krankheit verschlechtert, ist jedoch von Person zu Person unterschiedlich.

Es gibt verschiedene Behandlungen für die Krankheit. Diese Behandlungen helfen unterschiedlich gut, die Symptome zu lindern und das Fortschreiten der Krankheit zu verlangsamen. Sie haben aber auch unterschiedliche Nebenwirkungen.

Stellen Sie sich bitte weiter vor, dass Sie zusammen mit dem/der behandelnden Arzt/Ärztin eine Entscheidung treffen müssen, welche Behandlung angewendet werden soll. Um herauszufinden, welche Behandlung Sie bevorzugen, verwendet Ihre Arzt/Ärztin strukturierte Methoden.

Bitte geben Sie an, wie stark Sie folgenden Aussagen zustimmen:

	Stimme überhaupt nicht zu				Stimme voll und ganz zu
Ich bin mir bewusst, dass eine Entscheidung getroffen werden muss. * Pflichtfeld	○	○	○	◉	○
Ich bin mir bewusst, dass es unterschiedliche Behandlungsmöglichkeiten gibt. * Pflichtfeld	○	○	○	◉	○

Zurück

Weiter

Save & Return Later

Powered by REDCap

Bundesministerium
für Bildung
und Forschung

AAA

Methode 1

Hier sehen Sie zwei Eigenschaften von jeweils zwei verschiedenen Behandlungsmöglichkeiten für Ihre Krankheit im direkten Vergleich. Bitte wählen Sie bei jedem Vergleich die Option aus, für die Sie sich eher entscheiden würden. Auf Grundlage Ihrer Angaben, wird ihr Arzt/Ärztin Ihnen Behandlungsmöglichkeiten vorschlagen, die Ihren Präferenzen entsprechen.

Verabreichungsform: Sie müssen täglich eine Tablette einnehmen.

Symptome: Einige Symptome der Erkrankung bleiben bestehen, z. B. Gliederschmerzen.

Auswählen

Verabreichungsform: Sie müssen sich einmal wöchentlich ein Medikament in die Bauchdecke spritzen.

Symptome: Reduzierung der Symptome, sodass Sie im Alltag kaum beeinträchtigt sind.

Auswählen

Beide Optionen sind gleichwertig

Zurück

Save & Return Later

Bewerten Sie bitte Methode 1.
Bitte geben Sie an, inwiefern Sie den folgenden Aussagen zustimmen:

	Stimme überhaupt nicht zu				Stimme voll und ganz zu
Ich konnte zum Ausdruck bringen, welche Art der Behandlung ich bevorzuge.	○	○	○	○	○
Ich habe mit dieser Methode die unterschiedlichen Aspekte einer möglichen Behandlung gründlich abgewogen.	○	○	○	○	○
Ich finde diese Methode zu aufwändig.	○	○	○	○	○
Ich bin mit dieser Methode insgesamt zufrieden.	○	○	○	○	○
Wenn mein/e Arzt/Ärztin diese Methode bei der Suche nach einer passenden Behandlung benutzt, habe ich die Möglichkeit, meine Präferenzen aktiv einzubringen.	○	○	○	○	○
Ich wünsche mir, dass diese Methode im Gespräch mit dem/r behandelnden Arzt/Ärztin zur Anwendung kommt.	○	○	○	○	○

Zurück   Weiter

Save & Return Later

Methode 2

Bitte geben Sie jeder der folgenden Eigenschaften einer möglichen Behandlung Punkte, wobei die vergebene Punktzahl ausdrücken soll, wie wichtig Ihnen die jeweilige Eigenschaft ist. Für jede Eigenschaft reicht die Skala von 0 (unwichtig) bis 100 (sehr wichtig). Insgesamt (über alle Eigenschaften hinweg) müssen Sie genau 100 Punkte vergeben. Auf Grundlage Ihrer Angaben, wird ihr Arzt/Ärztin Ihnen Behandlungsmöglichkeiten vorschlagen, die Ihren Präferenzen entsprechen.

Möglichst einfache Anwendung der Behandlung.

30

Vermeiden von Symptomen.

50

Möglichst geringe Nebenwirkungen.

20

Verfügbare Punkte: **0**

Weiter

Zurück

Save & Return Later

Bewerten Sie bitte Methode 2.
Bitte geben Sie an, inwiefern Sie den folgenden Aussagen zustimmen:

	Stimme überhaupt nicht zu				Stimme voll und ganz zu
Ich konnte zum Ausdruck bringen, welche Art der Behandlung ich bevorzuge.	○	○	○	○	○
Ich habe mit dieser Methode die unterschiedlichen Aspekte einer möglichen Behandlung gründlich abgewogen.	○	○	○	○	○
Ich finde diese Methode zu aufwändig.	○	○	○	○	○
Ich bin mit dieser Methode insgesamt zufrieden.	○	○	○	○	○
Wenn mein/e Arzt/Ärztin diese Methode bei der Suche nach einer passenden Behandlung benutzt, habe ich die Möglichkeit, meine Präferenzen aktiv einzubringen.	○	○	○	○	○
Ich wünsche mir, dass diese Methode im Gespräch mit dem/r behandelnden Arzt/Ärztin zur Anwendung kommt.	○	○	○	○	○

Zurück Weiter

Save & Return Later

Powered by REDCap

Methode 3

Bitte wählen Sie aus, welche der drei folgenden Eigenschaften einer möglichen Behandlung Ihnen am wichtigsten ist, welche am unwichtigsten und welche weder am wichtigsten noch am unwichtigsten. Auf Grundlage Ihrer Angaben, wird ihr Arzt/Ärztin Ihnen Behandlungsmöglichkeiten vorschlagen, die Ihren Präferenzen entsprechen.

Möglichst einfache Anwendung der Behandlung.

- [x] Am wichtigsten
- [ ] Weder am wichtigsten noch am unwichtigsten
- [ ] Am unwichtigsten

Vermeiden von Symptomen.

- [ ] Am wichtigsten
- [x] Weder am wichtigsten noch am unwichtigsten
- [ ] Am unwichtigsten

Möglichst geringe Nebenwirkungen.

- [ ] Am wichtigsten
- [ ] Weder am wichtigsten noch am unwichtigsten
- [x] Am unwichtigsten

Weiter

Zurück

Save & Return Later

Bundesministerium für Bildung und Forschung

AAA

Bewerten Sie bitte Methode 3.
Bitte geben Sie an, inwiefern Sie den folgenden Aussagen zustimmen:

	Stimme überhaupt nicht zu				Stimme voll und ganz zu
Ich konnte zum Ausdruck bringen, welche Art der Behandlung ich bevorzuge.	○	○	○	○	○
Ich habe mit dieser Methode die unterschiedlichen Aspekte einer möglichen Behandlung gründlich abgewogen.	○	○	○	○	○
Ich finde diese Methode zu aufwändig.	○	○	○	○	○
Ich bin mit dieser Methode insgesamt zufrieden.	○	○	○	○	○
Wenn mein/e Arzt/Ärztin diese Methode bei der Suche nach einer passenden Behandlung benutzt, habe ich die Möglichkeit, meine Präferenzen aktiv einzubringen.	○	○	○	○	○
Ich wünsche mir, dass diese Methode im Gespräch mit dem/r behandelnden Arzt/Ärztin zur Anwendung kommt.	○	○	○	○	○

Zurück Weiter

Save & Return Later

Powered by REDCap

Bundesministerium für Bildung und Forschung

A A A

Methode 4

Stellen Sie sich bitte vor, es gibt folgende Behandlung für Ihre Krankheit, mit der Sie insgesamt noch 10 Jahre leben könnten:

Behandlung A:

- Sie müssen täglich eine Tablette einnehmen.
- Die Behandlung hat Nebenwirkungen, die Ihren Alltag beeinträchtigen, z. B. Erbrechen und Schwindel.
- Trotz der Behandlung bleiben einige Symptome der Erkrankung bestehen, z. B. Gliederschmerzen.

Würden Sie lieber 5 Jahr(e) in perfekter Gesundheit verbringen als 10 Jahre mit Behandlung A? Dafür würden Sie 5 Jahr(e) früher sterben.

Ja | Nein

Zurück

Save & Return Later

Powered by REDCap

Methode 4

Stellen Sie sich bitte vor, es gibt folgende Behandlung für Ihre Krankheit, mit der Sie insgesamt noch 10 Jahre leben könnten:

Behandlung B:

- **Sie müssen sich einmal wöchentlich ein Medikament in die Bauchdecke spritzen.**
- **Die Behandlung hat Nebenwirkungen, die Ihren Alltag beeinträchtigen, z. B. Müdigkeit und Übelkeit.**
- **Die Behandlung reduziert die Symptome der Erkrankung soweit, dass Sie im Alltag kaum beeinträchtigt sind.**

Würden Sie lieber 9 Jahr(e) in perfekter Gesundheit verbringen als 10 Jahre mit Behandlung B? Dafür würden Sie 1 Jahr(e) früher sterben.

Ja | Nein

Zurück

Save & Return Later

Bundesministerium für Bildung und Forschung

A A A

Methode 4

Stellen Sie sich bitte vor, es gibt folgende Behandlung für Ihre Krankheit, mit der Sie insgesamt noch 10 Jahre leben könnten:

Behandlung C:

- Sie müssen einmal pro Woche zu Ihrem Arzt fahren, um eine 30-minütige Infusion zu erhalten.
- Die Behandlung hat nur wenige Nebenwirkungen, die Ihren Alltag kaum beeinträchtigen, z. B. Mundtrockenheit.
- Die Behandlung reduziert die Symptome der Erkrankung soweit, dass Sie im Alltag kaum beeinträchtigt sind.

Würden Sie lieber 7 Jahr(e) in perfekter Gesundheit verbringen als 10 Jahre mit Behandlung C? Dafür würden Sie 3 Jahr(e) früher sterben.

Ja | Nein

Zurück

Save & Return Later

Bundesministerium für Bildung und Forschung

AAA

Bewerten Sie bitte Methode 4.
Bitte geben Sie an, inwiefern Sie den folgenden Aussagen zustimmen:

	Stimme überhaupt nicht zu				Stimme voll und ganz zu
Ich konnte zum Ausdruck bringen, welche Art der Behandlung ich bevorzuge.	○	○	○	○	○
Ich habe mit dieser Methode die unterschiedlichen Aspekte einer möglichen Behandlung gründlich abgewogen.	○	○	○	○	○
Ich finde diese Methode zu aufwändig.	○	○	○	○	○
Ich bin mit dieser Methode insgesamt zufrieden.	○	○	○	○	○
Wenn mein/e Arzt/Ärztin diese Methode bei der Suche nach einer passenden Behandlung benutzt, habe ich die Möglichkeit, meine Präferenzen aktiv einzubringen.	○	○	○	○	○
Ich wünsche mir, dass diese Methode im Gespräch mit dem/r behandelnden Arzt/Ärztin zur Anwendung kommt.	○	○	○	○	○

Zurück | Weiter

Save & Return Later

Powered by REDCap

Methode 5

Bitte stellen Sie sich vor, Sie würden aktuell mit folgender Behandlung ihrer Erkrankung leben:

Behandlung A:

- Sie müssen täglich eine Tablette einnehmen.
- Die Behandlung hat Nebenwirkungen, die Ihren Alltag beeinträchtigen, z. B. Erbrechen und Schwindel.
- Trotz der Behandlung bleiben einige Symptome der Erkrankung bestehen, z. B. Gliederschmerzen.

Es gibt eine andere Möglichkeit: Sie könnten in perfekter Gesundheit leben. Diese Alternative hat jedoch das Risiko, dass Sie sofort sterben könnten. Wie hoch dürfte dieses Risiko sein, damit Sie sich für die Alternative entscheiden? Oder würden Sie lieber in oben beschriebener Behandlung bleiben?

Ich möchte bei der beschriebenen Behandlung bleiben.

Ich möchte ein Risiko festlegen.

Powered by REDCap

Bundesministerium für Bildung und Forschung

A A A

Methode 5

Bitte stellen Sie sich vor, Sie würden aktuell mit folgender Behandlung ihrer Erkrankung leben:

Behandlung B:

- Sie müssen sich einmal wöchentlich ein Medikament in die Bauchdecke spritzen.
- Die Behandlung hat Nebenwirkungen, die Ihren Alltag beeinträchtigen, z. B. Müdigkeit und Übelkeit.
- Die Behandlung reduziert die Symptome der Erkrankung soweit, dass Sie im Alltag kaum beeinträchtigt sind.

Es gibt eine andere Möglichkeit: Sie könnten in perfekter Gesundheit leben. Diese Alternative hat jedoch das Risiko, dass Sie sofort sterben könnten. Wie hoch dürfte dieses Risiko sein, damit Sie sich für die Alternative entscheiden? Oder würden Sie lieber in oben beschriebener Behandlung bleiben?

Risiko für sofortiges Versterben beim Versuch, den perfekten Gesundheitszustand zu erreichen (%):

Min: 1% 66% Max: 99%

Ich möchte bei der beschriebenen Behandlung bleiben.

Ich bin bereit dieses Risiko einzugehen.

Zurück

Save & Return Later

Methode 5

Bitte stellen Sie sich vor, Sie würden aktuell mit folgender Behandlung ihrer Erkrankung leben:

Behandlung C:

- Sie müssen einmal pro Woche zu Ihrem Arzt fahren, um eine 30-minütige Infusion zu erhalten.
- Die Behandlung hat nur wenige Nebenwirkungen, die Ihren Alltag kaum beeinträchtigen, z. B. Mundtrockenheit.
- Die Behandlung reduziert die Symptome der Erkrankung soweit, dass Sie im Alltag kaum beeinträchtigt sind.

Es gibt eine andere Möglichkeit: Sie könnten in perfekter Gesundheit leben. Diese Alternative hat jedoch das Risiko, dass Sie sofort sterben könnten. Wie hoch dürfte dieses Risiko sein, damit Sie sich für die Alternative entscheiden? Oder würden Sie lieber in oben beschriebener Behandlung bleiben?

Risiko für sofortiges Versterben beim Versuch, den perfekten Gesundheitszustand zu erreichen (%):

Min: 1% 26% Max: 99%

Ich möchte bei der beschriebenen Behandlung bleiben.

Ich bin bereit dieses Risiko einzugehen.

Zurück

Save & Return Later

Bundesministerium für Bildung und Forschung

A A A

Bewerten Sie bitte Methode 5.
Bitte geben Sie an, inwiefern Sie den folgenden Aussagen zustimmen:

	Stimme überhaupt nicht zu				Stimme voll und ganz zu
Ich konnte zum Ausdruck bringen, welche Art der Behandlung ich bevorzuge.	○	○	○	○	○
Ich habe mit dieser Methode die unterschiedlichen Aspekte einer möglichen Behandlung gründlich abgewogen.	○	○	○	○	○
Ich finde diese Methode zu aufwändig.	○	○	○	○	○
Ich bin mit dieser Methode insgesamt zufrieden.	○	○	○	○	○
Wenn mein/e Arzt/Ärztin diese Methode bei der Suche nach einer passenden Behandlung benutzt, habe ich die Möglichkeit, meine Präferenzen aktiv einzubringen.	○	○	○	○	○
Ich wünsche mir, dass diese Methode im Gespräch mit dem/r behandelnden Arzt/Ärztin zur Anwendung kommt.	○	○	○	○	○

Zurück Weiter

Save & Return Later

Powered by REDCap

Vielen Dank für die Durchführung und Bewertung der Methoden.
Wir sind nach dieser Befragung auch schon fertig.

Bitte geben Sie an, inwieweit Sie folgenden Aussagen zustimmen:

	Stimme überhaupt nicht zu				Stimme voll und ganz zu
Mir ist es wichtig, zur Auswahl einer Therapie meine Präferenzen im Detail äußern zu können. * Pflichtfeld	○	○	○	○	○
Ich würde die Präferenzerhebung lieber in Ruhe bei mir Zuhause durchführen als bei meinem/r Arzt/Ärztin in einem 15-minütigen Gespräch. * Pflichtfeld	○	○	○	○	○
Mir haben die vorgestellten Methoden geholfen, mir über meine Präferenzen bewusst zu werden. * Pflichtfeld	○	○	○	○	○
Mir waren die Fragen in der Umfrage klar. * Pflichtfeld	○	○	○	○	○
Es gab Wörter oder Ausdrücke, die ich nicht verstanden habe. * Pflichtfeld	○	○	○	○	○
Es gab genügend Kontext, um die Fragen beantworten zu können. * Pflichtfeld	○	○	○	○	○

Können Sie sich vorstellen, dass zur Auswahl einer Therapie Ihre Präferenzen mithilfe einer der eben gezeigten Methoden erhoben werden?

* Pflichtfeld

Nein

Ja

Haben Sie noch irgendwelche Anmerkungen?

Erweitern

Zurück

Fertig

Save & Return Later
