# Supplementary material for: Assessing patient preferences for medical decision making - a comparison of different methods
Source: Front Digit Health. 2025 Nov 13;7:1641765. doi: 10.3389/fdgth.2025.1641765 (PMC12659191; doi:10.3389/fdgth.2025.1641765)
Supplement: Supplementary file 2 [file Datasheet2.pdf]

Translated questionnaire:

Introduction:

Dear participants,

In our research project, we aim to find out how patients and physicians can best be supported in making difficult medical decisions together.

On the following pages, we present five methods and ask you to apply them. After each method, you will have the opportunity to evaluate it by answering a few questions.

In addition, we will ask you for some information about yourself at the beginning and, at the end, for an overall assessment of the usefulness of these methods.

The survey is anonymous. It is not possible to draw any conclusions about your identity.

Many thanks for your support!

This survey is part of the project „EPAMeD – Ethics and Practice of Algorithm-Supported Decision-Making in Medicine“ about which you can find more information here. EPAMeD is funded by the Federal Ministry of Education and Research (BMBF) (grant number: 01GP2207).

If you have any questions about this survey, please contact …

For data protection matters, the Data Protection Officer of LMU is responsible (www.lmu.de/datenschutz).

Personal Questions:

Please first answer the following questions about yourself:

Age:
Required field

- 18–30
- 31–45
- 46–60
- 61–75
- 76+

Gender:
Required field

- Female
- Male
- Diverse

Do you speak German fluently?
Required field

- Yes
- No

What is the highest level of education you have completed?
Required field

- I have no school diploma
- Elementary school diploma
- Lower secondary school diploma (Hauptschule)
- Intermediate secondary school diploma (Mittlere Reife)
- General or subject-specific higher education entrance qualification (Abitur/Fachabitur)
- University degree

How often do you use a computer or smartphone?
Required field

- Less than once per month
- At least once per month
- At least once per week
- Daily

When was your last doctor’s visit?
Required field

- Less than 3 months ago
- 3–6 months ago
- 6–12 months ago
- More than 1 year ago

How would you rate your current state of health?
Required field

- Very good
- Good
- Moderate
- Poor
- Very poor

Do you have any chronic or acute illnesses?
Required field

- No, none
- Yes, acute
- Yes, chronic

Do you have a disability certificate?
Required field

- No
- Yes

Please think back to your past doctor’s visits: To what extent were you able to express your preferences when it came to making a medical decision?
Required field

Likert scale

- Not at all - - - - - Very much

Scenario „Medical Treatment Decision“

Please imagine that you have a serious illness that worsens over time. Without treatment, this illness will eventually cause you to hardly move, to need nursing support, and to die earlier. However, the speed at which the illness progresses differs from person to person.

There are different treatments for the illness. These treatments vary in how well they relieve symptoms and slow down the progression of the disease. However, they also have different side effects.

Now please imagine that you and your physician have to make a decision together about which treatment should be used. To find out which treatment you prefer, your physician uses structured methods.

Please indicate how strongly you agree with the following statements:

- I am aware that a decision has to be made.
  Required field

  Likert scale: Do not agree at all - - - - - Fully agree

- I am aware that there are different treatment options.

  Likert scale: Do not agree at all - - - - - Fully agree

Method evaluation:

Please evaluate Method x.
Please indicate to what extent you agree with the following statements:

- I was able to express which type of treatment I prefer.

  Likert scale: Do not agree at all - - - - - Fully agree

- With this method, I thoroughly weighed the different aspects of a possible treatment.

  Likert scale: Do not agree at all - - - - - Fully agree

- I find this method too time-consuming.

  Likert scale: Do not agree at all - - - - - Fully agree

- I am overall satisfied with this method.

  Likert scale: Do not agree at all - - - - - Fully agree

- If my doctor uses this method to find a suitable treatment, I have the opportunity to actively contribute my preferences.

  Likert scale: Do not agree at all - - - - - Fully agree

- I would like this method to be applied in conversations with my treating physician.

Likert scale: Do not agree at all - - - - - Fully agree

Questionaire evaluation:

Thank you very much for completing and evaluating the methods.
This concludes the survey.

Please indicate to what extent you agree with the following statements:

- It is important to me to be able to express my preferences in detail when choosing a therapy.
  Required field

  Likert scale: Do not agree at all - - - - - Fully agree

- I would prefer to complete the preference assessment quietly at home rather than with my doctor in a 15-minute conversation.
  Required field

  Likert scale: Do not agree at all - - - - - Fully agree

- The methods presented helped me to become aware of my preferences.
  Required field

  Likert scale: Do not agree at all - - - - - Fully agree

- The questions in the survey were clear to me.
  Required field

  Likert scale: Do not agree at all - - - - - Fully agree

- There were words or expressions that I did not understand.
  Required field

  Likert scale: Do not agree at all - - - - - Fully agree

- The questions provided enough context for me to answer them.
  Required field

  Likert scale: Do not agree at all - - - - - Fully agree

Can you imagine your preferences being assessed with one of the methods just presented when choosing a therapy?
Required field

- No
- Yes

Do you have any additional comments?
